# High Prevalence of Anthropometric-Only Obesity and Cardiometabolic Risk: Evidence from a Population-Based Study

**DOI:** 10.3390/nu18020229

**Published:** 2026-01-12

**Authors:** Vilma Kriaučionienė, Asta Raskilienė, Lina Šnipaitienė, Dalia Lukšienė, Abdonas Tamošiūnas, Ričardas Radišauskas, Vaiva Lesauskaitė, Janina Petkevičienė

**Affiliations:** 1 Health Research Institute, Faculty of Public Health, Lithuanian University of Health Sciences, Tilžės Str. 18, 47181 Kaunas, Lithuania; asta.raskiliene@lsmu.lt (A.R.); lina.snipaitiene@lsmu.lt (L.Š.); janina.petkeviciene@lsmu.lt (J.P.); 2 Department of Preventive Medicine, Faculty of Public Health, Lithuanian University of Health Sciences, Tilžės Str. 18, 47181 Kaunas, Lithuania; 3 Institute of Biological Systems and Genetic Research, Lithuanian University of Health Sciences, Eivenių Str. 4, 44307 Kaunas, Lithuania; 4 Institute of Cardiology, Lithuanian University of Health Sciences, Sukilėlių Av. 15, 50162 Kaunas, Lithuania; dalia.luksiene@lsmu.lt (D.L.); abdonas.tamosiunas@lsmu.lt (A.T.); ricardas.radisauskas@lsmu.lt (R.R.); vaiva.lesauskaite@lsmu.lt (V.L.)

**Keywords:** obesity phenotypes, cardiometabolic risk, diet, physical activity, urban population, Lithuania

## Abstract

**Background/Objectives**: The Lancet Commission proposes a new obesity definition that combines body mass index (BMI) with anthropometric measurements to distinguish adipose tissue excess more effectively. This study aims to determine the prevalence of obesity based on the new definition and to examine cardiometabolic risk factors and lifestyle habits across different obesity phenotypes in the urban population of Lithuania. **Methods**: This study was conducted among residents of Kaunas city from 2020 to 2024. A total of 3426 adults aged 25–69 years (57.1% of the random sample) were participated. Three individuals were excluded due to missing anthropometric data. Participants were categorized into three phenotypes: (1) no obesity (BMI < 30 kg/m^2^ and no or one elevated anthropometric measure, (2) anthropometric-only obesity (BMI < 30 kg/m^2^ and at least 2 elevated anthropometric measures), and (3) BMI-plus-anthropometric obesity (BMI ≥ 30 kg/m^2^ plus at least one elevated anthropometric measure or BMI ≥ 40 kg/m^2^). Standardized anthropometric, biochemical, and clinical measurements were collected, along with self-reported dietary habits and leisure-time physical activity. **Results**: Anthropometric-only obesity was highly prevalent, affecting 36.1% of males and 22.7% of females (*p* < 0.05). The prevalence of BMI-plus-anthropometric obesity was 24.1% among males and 21.4% among females. Individuals with anthropometric-only obesity had significantly higher odds of metabolic syndrome (OR 8.64; 95% CI 6.97–10.71), diabetes (OR 3.01; 95% CI 1.72–5.25), coronary heart disease (OR 1.48; 95% CI 1.12–1.97), and several lipid abnormalities compared with those without obesity. The highest cardiometabolic risk was observed in the BMI-plus-anthropometric obesity group. Greater adiposity was associated with higher intake of red meat, junk foods, and sugary drinks, while physical activity levels declined across obesity categories. **Conclusions**: Anthropometric-only obesity is a common and metabolically adverse phenotype that cannot be detected using BMI alone. A new obesity definition enhances identification of high-risk individuals and supports targeted prevention strategies.

## 1. Introduction

Obesity continues to be one of the main global health challenges and is closely linked to an increased risk of cardiometabolic disorders [1]. Despite various efforts to combat this issue, obesity rates are rising worldwide, reaching epidemic levels [2,3]. Several factors, including genetics, environment, and lifestyle choices, can contribute to body fat accumulation [3,4]. Traditionally, obesity in adults has been defined using the body mass index (BMI), calculated as weight in kilograms divided by height in meters squared [5]. While BMI is useful for population-level screening, it is not an accurate measure of adiposity, as it fails to consider body composition and fat distribution [6,7]. Recent recommendations from the Lancet Diabetes & Endocrinology Commission suggest a new approach to assessing obesity [8]. This updated framework combines BMI with anthropometric measurements, such as waist circumference, waist-to-height ratio, and waist-to-hip ratio, or direct measures of body fat, to distinguish adipose tissue excess more effectively. The framework also defines clinical obesity as excess adiposity accompanied by obesity-related organ dysfunction and/or physical limitations, and preclinical obesity, which is characterized by excess adiposity without any obesity-related organ dysfunction and/or physical limitations [8].

Several studies were conducted to determine the prevalence of obesity under the new definition and to evaluate the health outcomes associated with this new categorization. Data analysis from a population-based longitudinal cohort study conducted in the United States revealed a significantly higher prevalence of obesity due to the inclusion of individuals with anthropometric-only obesity [9]. The likelihood of organ dysfunction was greater for those with anthropometric-only obesity compared to individuals without obesity. An evaluation of longitudinal health outcomes showed that preclinical obesity was associated with an increased risk of developing diabetes and experiencing cardiovascular events [9]. The association between the new definition of obesity and mortality was analyzed using data from the UK Biobank. Both preclinical and clinical obesity were linked to a graded increase in all-cause mortality compared to individuals without obesity, with hazard ratios of 1.18 (95% confidence interval (CI), 1.14–1.23) and 1.56 (95% CI, 1.50–1.63), respectively [10]. In contrast, the traditional BMI-based classification indicated that being overweight was not associated with all-cause mortality. Similar patterns were observed for cardiovascular disease and cancer mortality [10]. These findings support the validity of the Lancet Commission classification, indicating that both categories of obesity—preclinical and clinical obesity—are related to an increased risk of mortality. However, clinical obesity identifies those at the highest cardiometabolic risk.

Lithuania has one of the highest rates of obesity and obesity-related health problems in Europe [11], highlighting the urgent need for new diagnostic approaches to identify high-risk individuals. When planning targeted interventions, which could help to reduce the burden of obesity-related diseases, it is essential to know the prevalence of obesity phenotypes based on the latest classification and to assess cardiometabolic risk linked to obesity categories. This study aims to determine the prevalence of obesity based on the new definition and to examine cardiometabolic risk factors and lifestyle habits across different obesity phenotypes in the urban population of Lithuania.

## 2. Materials and Methods

### 2.1. Study Design and Sample

The data were collected in a study titled “Chronic Diseases and Their Risk Factors in the Adult Population.” This study was conducted among residents of Kaunas, the second-largest city in Lithuania, from 2020 to 2024, with an interruption during the COVID-19 pandemic. A random sample of 6000 males and females aged 25 to 69 years was chosen from the Lithuanian population register, stratified by sex and age. Invitations were sent to the selected individuals to attend a health check-up at the Hospital of the Lithuanian University of Health Sciences Kaunas Clinics. A total of 3426 individuals participated in the study, resulting in a response rate of 57.1%. Three individuals were excluded from the analysis due to missing anthropometric data.

The study was approved by the Kaunas Regional Biomedical Research Ethics Committee (protocol number BE-2-49, issued on 5 June 2018). Written informed consent was obtained from all participants. No exclusion criteria were applied.

### 2.2. Measurements and Variables

All study participants completed a questionnaire that included the following sections: (1) social and demographic characteristics; (2) lifestyle factors, such as nutrition and physical activity; (3) health status and complaints; (4) diagnosis and management of risk factors and diseases; and (5) additional questions unrelated to this article. Participants were categorized into four age groups: 25–34, 35–44, 45–54, and 55–69 years old.

The questionnaire included questions about the respondent’s history of myocardial infarction, diabetes, and their use of antihypertensive, lipid-lowering, or glucose-lowering treatments.

Anthropometric measurements were conducted following a standardized protocol. Participants’ height was measured to the nearest 0.1 cm using a stadiometer, without shoes. Body weight was recorded to the nearest 0.1 kg with standardized medical scales while participants wore light indoor clothing and no shoes. BMI was calculated as weight divided by height squared (kg/m^2^). Waist circumference was measured at the midpoint between the lowest rib and the top of the iliac crest, to the nearest 0.1 cm. Hip circumference was measured at the widest point of the buttocks, also to the nearest 0.1 cm. A stretch-resistant tape was used for all circumference measurements.

Blood pressure was measured from the right brachial artery using a digital device while the participant was seated and rested for 5 min. Two consecutive measurements were taken, and the average of these two readings was used for analysis.

Blood samples for lipid and glucose measurements were collected in the morning after a fasting period of 12 h. The samples were obtained using venipuncture tubes to ensure they were free from hemolysis. All analyses were conducted in a certified laboratory utilizing the Selectra PRO XS biochemistry analyzer from EliTech Group (Spankeren, The Netherlands). For the enzymatic colorimetric analysis of total cholesterol, low-density lipoprotein (LDL) cholesterol, high-density lipoprotein (HDL) cholesterol, and triglycerides, reagents from EliTech Group B.V. were used. The analytes were detected directly by measuring the sample’s absorption at 500 nm. For plasma glucose measurement, the GLUCOSE OXIDASE-PAP/End Point Enzymatic PAP method was used. Continuous quality control of the measurements was maintained.

A resting electrocardiogram (ECG) was recorded using the 12 standard leads at a paper speed of 25 mm/s and an amplitude setting of 10 mm per 1 mV. Two independent experienced coders, both trained cardiologists, analyzed the ECG records using the Minnesota Code [12]. To assess ischemic heart pain, the Rose Angina Questionnaire was employed [13].

A food frequency questionnaire (FFQ) was used to evaluate dietary habits. The consumption frequency of 14 food categories, including vegetables, fruits, porridges, breakfast cereals, pasta, red meat, processed smoked red meat, processed cooked red meat, sweets and chocolate, confectionery, unhealthy snacks, fast food, convenience foods, and sweetened drinks were analyzed. Response options included the following categories: (1) ‘never’, (2) ‘1–3 times a month’, (3) ‘once a week’, (4) ‘several times a week’, (5) ‘daily’, and (6) ‘several times a day’. Physical activity was assessed by asking the question: ‘In your leisure time, how often do you engage in physical exercise for at least 30 min that makes you at least mildly short of breath or perspire?’ The possible answers were: ‘every day’, ‘4–6 times a week’, ‘2–3 times a week’, ‘once a week’, ‘once a month’, and ‘never’ coded from 1 to 7. Based on their responses, participants were categorized into two groups: (1) those physically active at least 2–3 times a week and (2) those physically active less often.

The diagnostic criteria for risk factors and diseases are outlined in Table 1.

Obesity was assessed using a new definition published in The Lancet Diabetes & Endocrinology [8]. Based on this new classification, individuals were divided into three groups: (1) ‘No obesity’ defined as having a BMI < 30 kg/m^2^ and no or one elevated anthropometric measure, such as waist circumference, waist-to-height ratio, or waist-to-hip ratio, (2) ‘Anthropometric-only obesity’ defined as having at least two elevated anthropometric measures with a BMI < 30 kg/m^2^, and (3) ‘BMI-plus-anthropometric obesity’ defined as having a BMI ≥ 30 kg/m^2^, along with at least one elevated anthropometric measure, or a BMI ≥ 40 kg/m^2^. In this study, all individuals with BMI ≥ 30 kg/m^2^ had at least one elevated anthropometric measure.

### 2.3. Statistical Analysis

The categorical variables were presented as percentages and compared using the χ^2^ test and the z-test with Bonferroni correction for multiple comparisons. To determine the overall prevalence of obesity phenotypes, the proportions were standardized by age, using the age structure of the Kaunas city population as the reference.

A logistic regression analysis was conducted to evaluate the association between obesity phenotypes and various risk factors or diseases. Separate models were computed for each risk factor and disease. An interaction analysis did not indicate significant sex- or age-specific differences in the direction of associations between obesity phenotypes and cardiometabolic risk factors. To adjust for sex and age, these variables were included as covariates in each logistic regression model.

Dietary patterns were obtained by exploratory factor analysis of 14 food categories. The data adjustment was verified using the Kaiser-Meyer-Olkin (KMO) measurement, which was 0.749, indicating good sample adequacy for factor analysis. Principal component analysis with Varimax rotation was used for factor extraction. Factor loadings greater than 0.4 were considered significant contributors to each factor. Five factors were extracted, accounting for 59.5% of the variability within the sample. The first factor was labeled ‘Junk Food and Sugary Drinks’, characterized by frequent consumption of unhealthy snacks, fast food, convenience foods, and sweetened beverages. The second factor, labeled ‘Red Meat’, was characterized by frequent intake of both unprocessed and processed red meat. The third factor was named ‘Sweets’ due to high loadings for sweets, chocolate, and confectionery. The fourth factor, labeled ‘Fruit and vegetables’, reflected frequent consumption of these foods. Finally, the fifth factor was named ‘Cereals’, as it showed high loadings for various porridges, breakfast cereals, and pasta.

The derived factors were used to create factor scores for individuals. These scores were then employed in linear regression analysis to examine the associations between dietary pattern (the dependent variable) and obesity phenotypes, which were coded as follows: (0) no obesity, (1) anthropometric-only obesity, and (2) BMI-plus-anthropometric obesity. Sex and age were included in each linear regression model.

Statistical analysis was performed using the statistical package IBM SPSS Statistics 27.0 (IBM Corp.: Armonk, NY, USA, released 2020). The criterion for statistical significance was set at *p* < 0.05.

## 3. Results

Table 2 presents characteristics of the study population by sex. Age distribution differed between sexes, with females being older than males (50.1 vs. 49.0 years, respectively (*p* = 0.008). Sex differences were observed in several obesity-related indicators: females were more likely than males to have a BMI < 25 kg/m^2^ (43.7% vs. 27.6%), whereas males more frequently fell into the overweight category (BMI 25–29 kg/m^2^: 45.1% vs. 31.3%). A higher proportion of females exceeded the sex-specific waist circumference cut-off. In contrast, males were more likely to have an elevated waist-to-height ratio and an increased waist-to-hip ratio (Table 2).

Cardiometabolic risk factors were unequally distributed between males and females. Arterial hypertension, hypertriglyceridemia, hyperglycemia, and metabolic syndrome were significantly more prevalent in males than in females. However, hypercholesterolemia was more common among females compared to males (61.6% vs. 53.7%, *p* < 0.001). In contrast, a higher proportion of males than females used statins to manage hypercholesterolemia (12.9% vs. 9.6%, *p* = 0.003). The differences in the prevalence of high LDL cholesterol and low HDL cholesterol between the sexes were not statistically significant. Furthermore, coronary heart disease, diagnosed using epidemiological criteria, was more prevalent among females (12.3% vs. 10.1%, *p* = 0.043), while diabetes was more frequently found among males (7.5% vs. 4.7%, *p* < 0.001). Regarding lifestyle indicators, leisure-time physical activity of at least 2–3 times per week was reported by a similar proportion of men and women (Table 2).

The distribution of respondents by the obesity phenotypes varied substantially among different age groups for both sexes (Table 3). In males, the proportion of those with normal weight declined sharply from 69.5% in the 25–34 age group to 13.8% in the 55–69 age group. This decline was accompanied by corresponding increases in both anthropometric-only obesity and BMI-plus-anthropometric obesity. A similar age-related pattern was observed among females, where the proportion classified as non-obese dropped from 76.8% in the 25–34 age group to 31.9% in the 55–69 age group. Additionally, anthropometric-only obesity increased consistently across all age groups for females, from 25–34 up to 55–69 years. The prevalence of BMI-plus-anthropometric obesity increased most in the 45–54 and 55–69 age groups.

Anthropometric-only obesity was more common in males than females across all age groups, except for those aged 25–34. The prevalence of BMI-plus-anthropometric obesity significantly differs between sexes only in the 45–55 age group.

**Figure 1:**
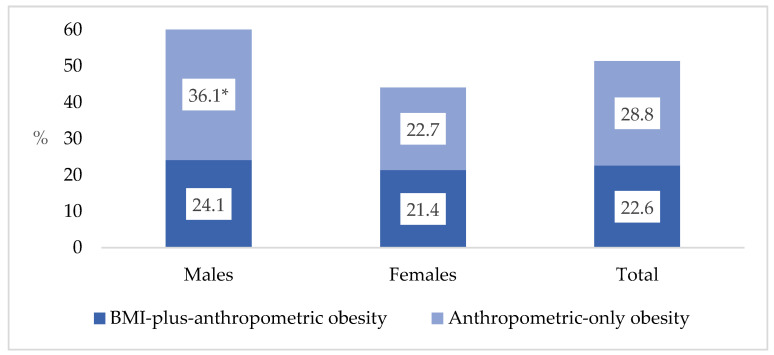
Age-standardized prevalence (%) of obesity phenotypes by sex. * *p* < 0.05 compared to females (z test with Bonferroni correction).

Figure 1 shows the age-standardized rates of obesity phenotypes based on the age structure of the Kaunas population in 2024. Almost half of the individuals (51.4%) had one of the obesity phenotypes. In general, males had a higher overall prevalence of obesity (60.1%) than females (44.1%), with a statistically significant difference noted in anthropometric-only obesity. However, there was no significant difference in the proportion of individuals with a BMI-plus-anthropometric obesity.

**Figure 2:**
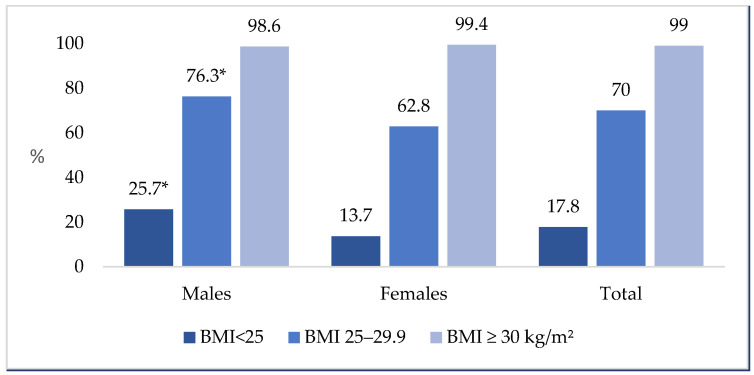
The proportion (%) of individuals with at least two elevated anthropometric indices based on BMI categories by sex. *p* < 0.001 between males and females (χ^2^ test); * *p* < 0.05 compared to females (z test with Bonferroni correction).

The proportion of individuals with at least two elevated anthropometric indices significantly increased across different BMI categories (Figure 2). Sex differences were found in the groups with BMI < 25 kg/m^2^ and BMI between 25 and 29.9 kg/m^2^ (*p* < 0.05), with men showing higher values than women. However, there were no significant sex differences in the group with a BMI ≥ 30 kg/m^2^. In fact, nearly all individuals with a BMI ≥ 30 kg/m^2^ (99%) had at least two elevated anthropometric indicators, demonstrating strong consistency between waist-based measures and BMI-defined obesity (Figure 2).

Both obesity categories were significantly associated with adverse cardiometabolic outcomes compared to individuals without obesity (reference group) (Table 4). Individuals with anthropometric-only obesity had higher odds of all risk factors, except for hypercholesterolemia. The likelihood of developing metabolic syndrome was 8.6 times greater for this group compared to the reference group. Additionally, the odds of experiencing diabetes and coronary heart disease were also higher among those with anthropometric-only obesity than in respondents without obesity.

The cardiometabolic risk was even more pronounced among individuals with BMI-plus-anthropometric obesity. This group had the highest odds of arterial hypertension, low HDL cholesterol, hypertriglyceridemia, and hyperglycemia. Furthermore, they had the greatest likelihood of metabolic syndrome (OR 33.28; 95% CI 25.99–42.62) and diabetes (OR 7.87; 95% CI 4.64–13.36).

The results of the linear regression analysis, which involved separate models for each dietary pattern adjusted for age and sex, indicated that some dietary patterns were significantly associated with obesity. In this study, obesity was categorized as follows: no obesity (0), anthropometric-only obesity (1), and BMI-plus-anthropometric obesity (2) (Table 5). The analysis revealed a positive association between obesity and higher intake of red meat, junk foods, and sugary drinks, suggesting that more frequent consumption of these food groups is linked to higher levels of obesity. Conversely, there was a negative association between obesity and the consumption of sweets and cereal products, indicating that as obesity levels increase, the intake of these foods tends to decrease. Additionally, the consumption of fruits and vegetables did not show a significant relationship with obesity phenotypes.

**Figure 3:**
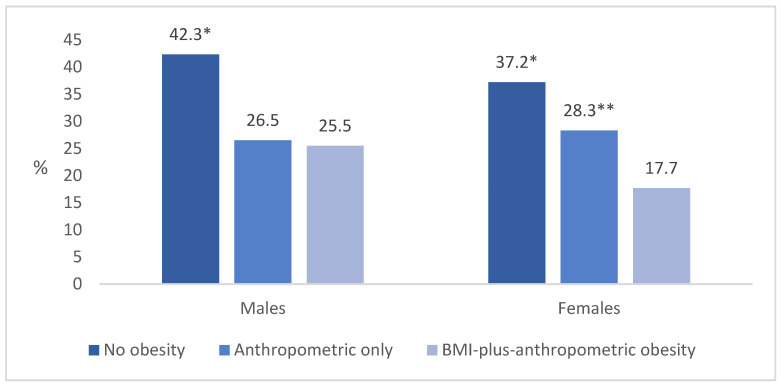
Age-standardized proportion (%) of males and females involved in leisure-time physical activity at least 2–3 times a week by obesity phenotypes. * *p* < 0.05 compared to anthropometric only and BMI-plus-anthropometric obesity groups; ** *p* < 0.05 compared to BMI-plus-anthropometric obesity group (z-test with Bonferroni correction).

In both males and females, the highest percentage of individuals engaging in leisure physical activity at least 2–3 times per week was found in the non-obesity category, with 42.3% of males and 37.2% of females participating (Figure 3). Among males, the proportion of those involved in leisure-time physical activity decreased by 1.6 times in both the anthropometric-only obesity group and the BMI-plus-anthropometric obesity group (*p* < 0.05). A similar trend was seen among females, where activity levels were 1.3 times lower in the anthropometric-only obesity group and 2.1 times lower in the BMI-plus-anthropometric obesity group compared to the non-obesity group (*p* < 0.05).

## 4. Discussion

This study provides evidence that the new obesity classification criteria proposed by the Lancet Diabetes & Endocrinology Commission in 2025 offer a more accurate assessment of adiposity-related risks than traditional BMI-based methods. Anthropometric-only obesity, a phenotype characterized by excess central adiposity despite a BMI of less than 30 kg/m^2^, was highly prevalent in our population, affecting 36.1% of males and 22.7% of females and representing a considerable subgroup at an elevated risk for cardiometabolic issues that would not be detected through BMI-only screening.

The differences in the prevalence of obesity phenotypes between sexes and across age groups observed in our study highlight the limitations of using BMI. Among males, the prevalence of anthropometric-only obesity increased sharply from 17.0% in the 25–34 age group to 52.2% in the 55–69 age group. In contrast, for females, this increase was more gradual, rising from 13.6% to 33.9% within the same age range. These sex differences are consistent with findings from several recent studies, which indicate that males tend to accumulate visceral fat earlier in adulthood and are more likely to have an unfavorable fat distribution at any given BMI compared to females [15,16]. Additionally, it has been noted that BMI often underestimates adiposity in older adults due to age-related changes in body composition [17,18]. A study conducted among a high-risk population of Mexican American individuals living in the United States found a very high prevalence of excess adiposity, with 94.1% of participants affected [19]. The research also revealed significant underdiagnosis of excess body fat in individuals whose BMI was below the obesity threshold. Notably, 87.9% of those with a BMI under 30 kg/m^2^ had excess body fat, highlighting the limitations of using BMI alone as an indicator of true adiposity. These findings support the Lancet Commission’s recommendation to redefine obesity based on excessive adiposity rather than body weight alone [8].

In our study, 25.7% of males and 13.7% of females with a BMI lower than 25 kg/m^2^ had at least two elevated anthropometric indices and were diagnosed with anthropometric-only obesity. Among individuals with a BMI between 25.0 and 29.9 kg/m^2^, the percentage with at least two elevated anthropometric measurements increased to 70%. International research increasingly supports the recognition of anthropometric-only obesity and normal-weight obesity as prevalent, clinically relevant phenotypes. The study conducted in the United States indicates that 25.9% of adults classified as non-obese by BMI meet anthropometric-only obesity [9]. In another US study, 33.1% of males and 51.9% of females had excess adiposity, being in the healthy BMI range 20–25 kg/m^2^ [20]. Similarly, research from Europe and Asia demonstrates that about 30% of adults with a normal BMI have excess abdominal adiposity and are at higher risk of conditions such as metabolic syndrome, insulin resistance, and dyslipidemia [21,22,23].

Our data show that metabolic impairments can start even before a person meets the BMI criteria for obesity. Compared with adults without obesity, those classified as having anthropometric-only obesity demonstrated a significantly higher prevalence of cardiometabolic problems. Specifically, the odds of metabolic syndrome were 8.6 times higher, while the odds of diabetes and coronary heart disease were 3.0 times and 1.5 times higher, respectively. Several metabolic abnormalities, including high LDL cholesterol, low HDL cholesterol, and hypertriglyceridemia, also indicate unfavorable metabolic risk. Notably, an inverse association with hypercholesterolemia was observed in the BMI-plus-anthropometric obesity group. We hypothesize that this finding likely reflects the effects of pharmacological lipid-lowering treatment rather than a genuinely favorable lipid profile. Individuals with this phenotype demonstrated a higher prevalence of statin use, which may have contributed to lower total cholesterol levels at the time of examination. Since hypercholesterolemia was defined based on current lipid measurements, cross-sectional analyses may underestimate prior dyslipidemia in treated individuals. Similar patterns have been reported in populations with high rates of statin use [24].

Many studies have established a link between ageing and cardiometabolic dysfunction. This relationship is influenced by age-related metabolic changes, oxidative stress, chronic inflammation, and lifestyle factors [25]. Additionally, sex plays a significant role in the risk of developing cardiometabolic syndrome. Hormonal differences, variations in fat distribution (with women tending to have more visceral fat and men more subcutaneous fat), and genetic factors contribute to different risk profiles for men and women [26]. However, interaction analyses conducted in our study did not indicate significant sex- or age-specific differences in the direction of associations between obesity phenotypes and cardiometabolic risk factors. Consequently, sex and age were included in all regression models assessing the associations between cardiometabolic risk and obesity phenotypes to account for these variables.

Recent large-scale research from 91 countries has similarly reported that individuals with normal BMI but abdominal obesity face significant metabolic disadvantages, such as higher triglyceride levels, impaired glucose regulation, and greater odds of metabolic syndrome compared to adults with normal waist circumference [27]. A meta-analysis showed that central obesity markers are more reliable predictors of developing type 2 diabetes than BMI alone [28]. These findings align with a study conducted in the United States, which revealed that individuals with preclinical obesity, characterized as excess abdominal fat without clear organ dysfunction, have a significantly higher risk of developing diabetes and experiencing cardiovascular events [9]. Twenty-year follow-up data from the ATTICA study demonstrated that central adiposity was the strongest predictor of long-term cardiovascular events, irrespective of BMI category [29]. Furthermore, recent UK Biobank data showed that central adiposity substantially increases long-term mortality risk, indicating that visceral fat accumulation, not overall weight, is the primary contributor to cardiometabolic outcomes [10]. These findings emphasize the importance of identifying abdominal obesity at much earlier stages, before metabolic dysfunction becomes clinically apparent.

The anthropometric measures used in this study—waist circumference, waist-to-height ratio, and waist-to-hip ratio—reflect excess central and visceral fat. These indicators are strongly associated with metabolically active fat deposits that contribute to insulin resistance, dyslipidemia, systemic inflammation, and abnormal fat accumulation in other areas of the body. Unlike BMI, which measures total body mass, waist-based indicators focus on the distribution of adiposity. This allows for better identification of individuals with excess abdominal fat, even if their overall body weight is normal or moderately elevated. This is consistent with evidence showing that the accumulation of visceral fat is a significant factor in cardiometabolic risk, independent of BMI [8,27,28,29]. Some individuals with a BMI greater than 30 kg/m^2^ do not show any cardiometabolic abnormalities, which means they can be categorized as having metabolically healthy obesity. Therefore, incorporating additional anthropometric measures alongside BMI could lead to a more accurate identification of those at an increased risk of metabolic complications [30].

Nutrition is a major modifiable factor that can be targeted to address the rising prevalence of obesity and metabolic disorders. In our study, dietary patterns varied across different adiposity phenotypes. Higher levels of adiposity were linked to increased consumption of red meat, junk foods, and sugary drinks, whereas inverse associations were observed for sweets and cereals. No significant differences were found in the intake of fruits and vegetables. These findings suggest that adults with higher levels of adiposity tend to have less favorable dietary behaviors. The observed inverse association between refined carbohydrates intake and adiposity can be explained in several ways. Individuals with obesity are more likely—often unintentionally—to under-report their intake of high-calorie or “unhealthy” foods, such as sweets, pastries, and sugary cereals [31]. Many people with obesity are already trying to lose weight or improve their diet by cutting back on sweets and refined cereals or replacing cereals with higher-protein or lower-carb options. Additionally, individuals who have developed obesity-related health conditions may consciously limit their intake of sugary foods and processed cereals to reduce the risk of diabetes and other metabolic complications. A study conducted in the United States revealed that individuals who were overweight and obese tended to obtain less energy from total carbohydrates and more from total fat [32].

Global evidence indicates that poor dietary quality significantly contributes to metabolic dysfunction and the burden of chronic diseases [33]. Research on normal-weight obesity supports that dietary quality plays a crucial role in fat accumulation, even among individuals with a normal BMI. The PACE study in Brazil demonstrated that children with a higher consumption of processed and ultra-processed foods showed increases in their body fat during adolescence [34]. A study conducted among Thai women found that normal-weight obesity was linked to dietary patterns that included a high frequency of ultra-processed foods and sugary beverages [22]. Another study identified consumption of sweet bakery items, sugary drinks, fast food, and irregular meal patterns as predictors of normal-weight obesity among females [35]. Bellissimo et al. observed that higher diet quality was associated with decreased total and visceral fat [36]. Additionally, longitudinal analyses from the China Health and Nutrition Survey provide further support, demonstrating that maintaining healthy dietary patterns can markedly decrease the risk of adiposity [37].

Our study showed that physical activity decreases as adiposity increases. Among males, the percentage of those engaging in regular leisure-time activity (at least 2–3 times per week) decreased from 42.3% in the non-obese group to 26.5% in the anthropometric-only phenotype and to 25.5% in the BMI-plus-anthropometric obesity group. A similar trend was observed among females, with activity levels declining from 37.2% to 28.3% and 17.7%, respectively. These findings are consistent with observations that individuals with normal-weight obesity have lower aerobic capacity and reduced physical fitness compared to normal-weight lean adults [36,38]. Research using large-scale accelerometry indicated that an increase in sedentary time and a reduction in moderate-to-vigorous physical activity are strongly associated with higher insulin resistance, adverse lipid profiles, and elevated mortality risk [39]. Experimental studies have shown that prolonged sitting impairs mitochondrial function, lowers oxidative capacity, and increases pro-inflammatory signaling [40]. In contrast, structured exercise improves insulin sensitivity, reduces visceral and liver fat, and enhances lipid metabolism [41].

Dietary habits and patterns of physical activity vary across different adiposity phenotypes and contribute to metabolic risk. Emphasizing the importance of increasing physical activity and improving physical fitness, along with promoting healthy eating habits, may be crucial for reducing risk in individuals with anthropometric-only obesity. Further research is needed to determine whether achieving high dietary quality and increased levels of physical activity effectively help individuals with anthropometric-only obesity reduce fat mass and lower long-term cardiometabolic risk.

### Limitations and Strengths

This study has several limitations that should be considered when interpreting the findings. First, the study sample comprised adults from Kaunas, Lithuania’s second-largest city. Therefore, an urban setting may limit the generalizability of the findings regarding the prevalence of obesity phenotypes to rural populations, where lifestyle patterns and socioeconomic factors can differ. Nevertheless, we believe that the associations between obesity phenotypes and cardiometabolic risk are unlikely to vary significantly across different settings. Second, although we collected a medical history of complaints and illnesses and performed many measurements, we lacked sufficient information on some organ dysfunction to distinguish between preclinical and clinical obesity as recommended by the Lancet Diabetes & Endocrinology Commission experts. Third, information on dietary habits and physical activity was self-reported, which increases the possibility of recall and social desirability bias. Finally, the cross-sectional study design limits causal interpretation of associations between lifestyle factors, obesity phenotypes and metabolic disorders.

Despite these limitations, the study has several important strengths. A key advantage is the use of a randomly selected population-based sample, which enhances the representativeness of the findings and reduces the likelihood of selection bias. Standardized clinical and anthropometric measurements performed by trained personnel and the availability of validated biochemical markers contribute to high data accuracy. Another notable strength is the application of updated Lancet Clinical Obesity criteria, which allowed us to identify obesity phenotypes that remain undetected when BMI alone is used. The ability to link these phenotypes with a broad set of lifestyle indicators, including diet and physical activity, provides additional insight into behavioral correlates of adiposity and enriches the interpretation of cardiometabolic patterns.

## 5. Conclusions

In summary, this study demonstrates that obesity, defined by recently proposed adiposity-based criteria, is highly prevalent in a Lithuanian urban population and is strongly associated with adverse cardiometabolic profiles. Anthropometric-only obesity was common, particularly among males and older adults, and was already accompanied by significant metabolic abnormalities despite BMI values that did not meet obesity criteria. Individuals with combined BMI and waist-based obesity measures showed the highest burden of hypertension, dyslipidemia, hyperglycemia, and metabolic syndrome, reflecting a clear progression of risk across phenotypes. Although lifestyle factors partly explained differences in adiposity and are well recognized as contributors to cardiometabolic disease pathogenesis, they did not diminish the central role of abdominal adiposity in shaping cardiometabolic risk. These findings highlight the need to incorporate waist-based indicators into routine clinical evaluation and support early, targeted interventions aimed at preventing the progression from excess adiposity to clinically evident cardiometabolic disease.

## Figures and Tables

**Table 1:** The diagnostic criteria for risk factors and diseases.

Risk Factor or Disease	Diagnostic Criteria
Increased waist circumference	≥102 cm for males, ≥88 cm for females
Increased waist-to-height ratio	≥0.5
Increased waist-to-hip ratio	≥0.9 for males, ≥0.85 for females
Arterial hypertension	Systolic blood pressure (BP) > 140 mmHg and/or diastolic BP > 90 mmHg or BP < 140/90 mmHg using antihypertensive medication for the last two weeks before examination.
Hypercholesterolemia	≥5.2 mmol/L
High LDL cholesterol	≥3 mmol/L
Low HDL cholesterol	<1.03 mmol/L for males, <1.29 mmol/L for females
Hypertriglyceridemia	≥1.7 mmol/L
Hyperglycemia	≥5.6 mmol/L
Metabolic syndrome	Waist circumference ≥ 94 cm for males and ≥80 cm for females plus any two of the following four factors: raised triglycerides level (≥1.7 mmol/L); reduced HDL lipoprotein cholesterol level (for men < 1.03 mmol/L and for women < 1.29 mmol/L); raised blood pressure (systolic BP ≥ 130 mm Hg or diastolic BP ≥ 85 mm Hg, or treatment of previously diagnosed hypertension); raised fasting plasma glucose (≥5.6 mmol/L or previously diagnosed type 2 diabetes)—criteria of International Diabetes Federation [12].
Diabetes	Diabetes was defined as having a fasting blood glucose level of at least 7 mmol/L, the use of blood glucose-lowering medication, or a self-reported diagnosis received from a physician.
Coronary heart disease (CHD)	The epidemiological criteria for the identification of CHD at the time of the health check according to the priority are: (1) exposure to myocardial infarction and/or ischemic changes on the ECG, as assessed by Minnesota codes 1–1, 1–2 [13]; (2) exertional angina pectoris identified using G. Rose questionnaire [14]; (3) ischemic ECG changes assessed using the following Minnesota codes: 1–3, 4–1, 4–2, 4–3, 5–1, 5–2, 5–3, 6–1, 6–2, 7–1, 8–3.

Abbreviations: LDL—low-density lipoproteins; HDL—high-density lipoproteins; ECG—electrocardiogram.

**Table 2:** Characteristics of the study population, n (%).

Characteristics	Males n = 1522	Females n = 1901	*p*-Value
Age (years), mean (SD)	49.0 (11.5)	50.1 (11.0)	0.008
Age groups (years)			0.009
25–34	200 (13.1) *	198 (10.4)	
35–44	387 (25.4)	437 (23.0)	
45–54	341 (22.4) *	483 (25.4)	
55–69	594 (39.1)	783 (41.2)	
BMI categories (kg/m^2^)			<0.001
<25	420 (27.6) *	830 (43.7)	
25–29	687 (45.1) *	596 (31.3)	
≥30–39	385 (25.3)	431 (22.7)	
≥40	30 (2.0)	44 (2.3)	
Waist circumference (cm)			<0.001
<102 for males, <88 for females	931 (61.2)	1007 (53.0)	
≥102 for males, ≥88 for females	591 (38.8)	894 (47.0)	
Waist-to-height ratio			<0.001
<0.5	363 (23.9)	751 (39.5)	
≥0.5	1159 (76.1)	1150 (60.5)	
Waist-to-hip ratio			<0.001
<0.9 for males, <0.85 for females	425 (27.9)	1173 (61.7)	
≥0.9 for males, ≥0.85 for females	1097 (72.1)	728 (38.3)	
Arterial hypertension			<0.001
No	579 (38.1)	1127 (59.3)	
Yes	942 (61.9)	772 (40.7)	
Total cholesterol level (mmol/L)			<0.001
<5.2	703 (46.3)	730 (38.4)	
≥5.2	814 (53.7)	1169 (61.6)	
LDL cholesterol level (mmol/L)			0.053
<3	743 (49.0)	867 (45.7)	
≥3	774 (51.0)	1032 (54.3)	
Patients taking statins	196 (12.9)	183 (9.6)	0.003
HDL cholesterol level (mmol/L)			0.843
≥1.03 for males, ≥1.29 for females	900 (59.3)	1133 (59.7)	
<1.03 for males, <1.29 for females	617 (40.7)	766 (40.3)	
Triglyceride level (mmol/L)			<0.001
<1.7	1040 (68.6)	1492 (78.6)	
≥1.7	477 (31.4)	407 (21.4)	
Glucose level (mmol/L)			<0.001
5.6	777 (51.3)	1329 (70.1)	
≥5.6	739 (48.7)	568 (29.9)	
Metabolic syndrome			<0.001
No	744 (49.0)	1142 (60.2)	
Yes	773 (51.0)	755 (39.8)	
Diabetes			<0.001
No	1408 (92.5)	1812 (95.3)	
Yes	114 (7.5)	89 (4.7)	
Coronary heart disease			0.043
No	1369 (89.9)	1668 (87.7)	
Yes	153 (10.1)	233 (12.3)	
Leisure-time physical activity			0.639
At least 2–3 days a week	455 (30.4)	558 (29.6)	
Less often	1042 (69.6)	1324 (70.4)	

* *p* < 0.05 (z-test with Bonferroni correction). Abbreviations: BMI—body mass index; LDL—low-density lipoproteins; HDL—high-density lipoproteins.

**Table 3:** Distribution (%) of males and females by obesity phenotypes and age.

Sex	Obesity Phenotypes	Age Groups (Years)				*p*-Value
		25–34	35–44	45–54	55–69	
Males		n = 200	n = 387	n = 341	n = 594	<0.001
	No obesity	69.5 *	45.2 *^#^	23.2 *^#^	13.8 *^#^	
	Anthropometric only obesity	17.0 *	33.1 *^#^	46.9 ^#^	52.2 ^#^	
	BMI-plus-anthropometric obesity	13.5 **	21.7 ***	29.9 ^#^	34.0	
Females		n = 198	n = 437	n = 483	n = 783	<0.001
	No obesity	76.8 *	65.2 *	52.0 *	31.9 *	
	Anthropometric only obesity	13.6 **	17.9	24.4	33.9 *	
	BMI-plus-anthropometric obesity	9.6 **	16.9	23.6	34.2 *	

* *p* < 0.05 compared to all other age groups; ** *p* < 0.05 compared to 45–54 and 55–69 age groups; *** *p* < 0.05 compared to 55–69 age group; ^#^ *p* < 0.05 compared to females (z-test with Bonferroni correction); BMI—body mass index.

**Table 4:** Odds ratios of risk factors and diseases by obesity phenotypes (logistic regression analysis).

Risk Factor or Disease	Obesity Phenotypes			
	Anthropometric Only Obesity		BMI-Plus-Anthropometric Obesity	
	OR (95% CI)	*p*-Value	OR (95% CI)	*p*-Value
Arterial hypertension	1.98 (1.64–2.39)	<0.001	6.58 (5.31–8.14)	<0.001
Hypercholesterolemia	1.13 (0.95–1.35)	0.183	0.81 (0.67–0.97)	0.024
High LDL cholesterol	1.29 (1.09–1.54)	0.004	0.90 (0.75–1.08)	0.245
Low HDL cholesterol	2.20 (1.83–2.64)	<0.001	4.46 (3.68–5.40)	<0.001
Usage of statins	2.08 (1.49–2.89)	<0.001	2.81 (2.02–3.91)	<0.001
Hypertriglyceridemia	2.60 (2.10–3.22)	<0.001	3.96 (3.19–4.91)	<0.001
Hyperglycemia	1.70 (1.41–2.05)	<0.001	3.59 (2.96–4.36)	<0.001
Metabolic syndrome	8.64 (6.97–10.71)	<0.001	33.28 (25.99–42.62)	<0.001
Diabetes	3.01 (1.72–5.25)	<0.001	7.87 (4.64–13.36)	<0.001
Coronary heart disease	1.48 (1.12–1.97)	0.007	1.87 (1.41–2.48)	<0.001

A separate logistic regression model was computed for each risk factor, adjusting for age and sex. The reference group consisted of participants without obesity. Abbreviations: OR—odds ratio; CI—confidence intervals; LDL—low density lipoproteins; HDL—high density lipoproteins.

**Table 5:** Associations between dietary patterns and obesity phenotypes (linear regression analysis).

Dietary Patterns	Unstandardized Coefficients	95% CI	*p*-Value	Standardized Coefficients	R^2^
Junk Food and Sugary Dinks	0.057	0.020–0.094	0.002	0.046	0.309
Red Meat	0.183	0.141–0.225	<0.001	0.147	0.094
Sweets	−0.145	−0.189–−0.101	<0.001	−0.117	0.015
Fruit and Vegetables	−0.030	−0.073–0.014	0.187	−0.024	0.022
Cereals	−0.075	−0.119–−0.030	0.001	−0.060	0.016

A separate linear regression model was computed for each dietary pattern, adjusting for age and sex. Abbreviations: CI—confidence intervals.

## Data Availability

The data presented in this study are available on request from the corresponding author (Public data sharing is restricted due to ethical considerations, as specified in the Bioethics Committee approval, which does not permit open access to individual-level data).

## Abbreviations

The following abbreviations are used in this manuscript:

BMI	Body mass index
WC	Waist circumference
WHR	Waist-to-hip ratio
WHtR	Waist-to-height ratio
HDL	High-density lipoprotein cholesterol
LDL	Low-density lipoprotein cholesterol
BP	Blood pressure
ECG	Electrocardiogram
OR	Odds ratio
CI	Confidence interval
SD	Standard deviation
KMO	Kaiser–Meyer–Olkin measure
CHD	Coronary heart disease
